# Sex-Related Differences in Patients with Mitral Regurgitation Undergoing Mitral Valve Surgery: A Propensity Score-Matched Study

**DOI:** 10.3390/jcm14093054

**Published:** 2025-04-28

**Authors:** Edouard Long, Omar Chehab, Tanisha Rajah, Roberta Dunn, Vitaliy Androshchuk, Joshua Wilcox, Harminder Gill, Vassilios Avlonitis, Paolo Bosco, Gianluca Lucchese, Tiffany Patterson, Simon Redwood, Ronak Rajani

**Affiliations:** 1 Institute of Cardiovascular Science, University College London, London W1W 7TS, UK; 2 Faculty of Life Sciences and Medicine, King’s College London, London SE1 9NH, UK; 3 Department of Cardiology, St Thomas’ Hospital, London SE1 9RT, UK; omarchehab@doctors.org.uk (O.C.);; 4 School of Medical Sciences, University of Birmingham, Birmingham B15 2TT, UK; 5 Department of Cardiac Surgery, St Thomas’ Hospital, London SE1 9RT, UK

**Keywords:** mitral regurgitation, cardiac surgery, sex differences, mitral valve, echocardiography, propensity score

## Abstract

**Background/Objectives**: Sex-related differences in the presentation and outcomes of patients with mitral regurgitation (MR) undergoing mitral valve (MV) surgery remain unclear. We aimed to identify these differences to inform personalized management. **Methods:** A total of 143 consecutive patients undergoing surgery for MR between 2017 and 2018 were stratified by sex and assessed for differences in characteristics. We performed 1:1 propensity score matching (PSM) by sex, with baseline characteristics as covariates, yielding 38 comparable pairs which were analyzed for differences in all-cause mortality and post-operative length of stay (LOS). **Results:** Females (*n* = 67) were more symptomatic (NYHA Class ≥ 3: 73% vs. 45%, *p* < 0.001), had higher logistic EuroSCORE (5.5 vs. 3.9, *p* = 0.006), had more urgent operations (25% vs. 11%, *p* = 0.020), MV replacements (28% vs. 11%, *p* = 0.007), and secondary MR (43% vs. 16%, *p* < 0.001). Females had significantly smaller end-diastolic and end-systolic left ventricular (LV) diameters, though indexed diameters showed no significant differences. After PSM, females had significantly longer LOS (7 days vs. 9 days, *p* = 0.033) and no differences in long-term mortality (hazard ratio [HR]: 1.25, 95% confidence interval [CI]: 0.34–4.76, *p* = 0.7, median follow-up: 6.67 years). An indexed LV end-systolic diameter (LVESDi) > 19 mm/m^2^ yielded greater specificity (46.0% vs. 26.7%) and comparable sensitivity (69.4% vs. 69.2%) to LVESD > 40 mm. In subgroup analyses, female patients undergoing concomitant tricuspid intervention (HR: 6.80 [1.63–37.92], *p* < 0.01) or urgent operation (HR: 4.85 [1.08–21.06], *p* = 0.04) had worse prognoses than males. **Conclusions:** Females undergoing MV surgery for MR had more symptoms, higher surgical risk, and longer LOS, but similar mortality compared to males. However, concomitant tricuspid intervention and urgent operations were associated with higher mortality. Our results add to the growing body of evidence that current non-indexed LV diameter thresholds may not adequately account for sex differences.

## 1. Introduction

There has been an increasing awareness of sex-related differences in the presentation and outcomes of both primary and secondary mitral regurgitation (MR). It has been shown that women with MR are less likely to undergo multidisciplinary evaluation [1], have lower rates of mitral valve (MV) intervention [2], are more likely to develop heart failure [3], and are less likely to receive concomitant tricuspid valve repair (TVr) [4] for severe tricuspid regurgitation (TR).

Although the cause for this is undoubtedly multifactorial, it has been proposed that these discrepancies may in part be due to intrinsic differences in cardiac anatomy which are not accounted for using non-adjusted echocardiography measurements [2,5,6]. The recognition and implementation of sex-specific thresholds for intervention have been adopted in international guidelines for aortic regurgitation [7,8] but this is not the case for MR despite the evidence that females present with smaller non-indexed left ventricular (LV) dimensions. In the context of primary MR, this means that women are therefore less likely to meet Class I surgical thresholds (left ventricular end-systolic diameter [LVESD] > 40 mm) for intervention [7,8].

It has been noted that there is a lack of studies investigating sex-related differences in MV surgery which propensity match patients based on preoperative characteristics and comorbidities [9], complicating the true elucidation of the role sex may play in outcomes. Therefore, we sought to investigate sex-related differences in the presentation, intervention, and outcomes of patients with primary and secondary MR in a large tertiary center using robust propensity-score matching. We also aimed to investigate whether using non-indexed LV diametric assessment results in sex differences and compare it to LV diameters indexed to body surface area (BSA).

## 2. Materials and Methods

### 2.1. Study Population

Consecutive patients undergoing MV surgery between 2017 and 2018 for the treatment of primary and secondary MR at a tertiary cardiac center, St Thomas’ Hospital, (Guy’s and St Thomas’ NHS Foundation Trust, London, UK), were included in this retrospective analysis. Exclusion criteria were (1) diagnosis of infective endocarditis; (2) concomitant bypass surgery; (3) missing baseline echocardiography measurements. All patients underwent comprehensive evaluation by a multidisciplinary heart team prior to treatment. Surgical MV interventions were performed using a standard median sternotomy approach. Ethical approval for this study was not required as it was performed as part of an outcome audit.

Baseline clinical, echocardiographic, and peri-operative characteristics were collected from electronic health records and collated into an electronic database. All patients had pre-operative echocardiography, performed by British Society of Echocardiography-accredited operatives according to European guidelines [10], within 6 months pre-operation.

### 2.2. Outcomes

The primary outcome was all-cause mortality and a secondary endpoint of post-operative length of hospital stay (LOS) was also assessed.

### 2.3. Statistical Analysis

Numerical data are presented as median (interquartile range [IQR]) and categorical data are presented as number (percentage). Normality of variables was assessed visually using Q-Q plots and quantitatively with the Shapiro–Wilk test. Subsequently, between-group comparisons were conducted using the Mann–Whitney U test for numerical data and using the chi-squared or Fisher’s test for categorical data, where indicated.

Propensity score matching (PSM) was used to control for differences in the patients’ baseline characteristics. A logistic regression model was used to calculate propensity scores using the following characteristics: age, body mass index, diabetes, hypertension, chronic obstructive pulmonary disease, prior myocardial infarction, prior cerebrovascular event, prior cardiac surgery, estimated glomerular filtration rate, logistic EuroSCORE, left ventricular ejection fraction (LVEF), left-ventricular indexed end-systolic and end-diastolic diameter, TR ≥ 2+, MR etiology, New York Heart Association (NYHA) class ≥ 3, MV repair vs. replacement, and concomitant TV repair. Male and female patients were matched in a 1:1 ratio using nearest neighbor PSM with a fixed caliper width of 0.02. After matching, balance within matched pairs was examined using standardized differences in covariate means and proportions. Cumulative survival rates were assessed using the Kaplan–Meier method and between-group differences in survival were made using a Cox regression model with a robust sandwich variance estimate to account for paired data. Survival was also assessed in the unmatched cohort using the log-rank test. Receiver operating characteristic (ROC) curve analysis was then performed to compare the sensitivity and specificity of the traditionally used threshold of LVESD (40 mm) to LVESDi dichotomized according to the median value in the cohort (19 mm/m^2^).

In a supplementary analysis, we investigated subgroups of interest (MV repair vs. replacement, concomitant TV intervention, urgent vs. elective operation) separately in males and females for differences in the primary and secondary outcomes. Firth’s penalized Cox regression was used to assess all-cause mortality to mitigate any potential bias arising from low event rates, and LOS was assessed using a linear regression model. As we identified differences in outcomes after concomitant TV intervention, we sought to investigate this further by looking at baseline right ventricular (RV) function on TTE using the ratio of tricuspid annular plane systolic excursion (TAPSE) to pulmonary artery systolic pressure (PASP), an index of right ventricular-to-pulmonary artery coupling which provides a comprehensive measure of RV function and which we have previously identified as an independent predictor of survival in this cohort of patients [11].

Proportional hazards, non-linearity, and influential observations were assessed using Schoenfeld residuals, Martingale residuals, and deviance residuals, respectively. All analyses were performed using R version 4.4.0 (R Foundation for Statistical Computing, Vienna, Austria). Statistical significance was defined as *p* < 0.05 for all statistical tests.

## 3. Results

### 3.1. Patient Charicteristics

**Figure 1:**
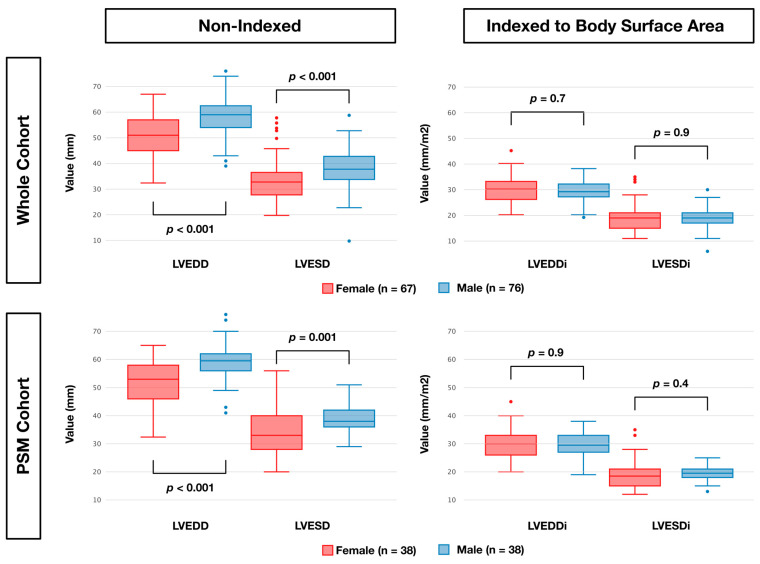
Comparison of indexed to non-indexed left ventricular diameters by sex in whole and PSM cohorts. Diameters are indexed to body surface area. Pairwise tests by Mann–Whitney U test. Outliers are shown as dots. Abbreviations: PSM, propensity score-matched; LVEDD, left ventricular end-diastolic diameter, LVEDDi, left ventricular end-diastolic diameter indexed to body surface area; LVESD, left ventricular end-systolic diameter; LVESDi, left ventricular end-systolic diameter indexed to body surface area.

A total of 143 patients met the inclusion criteria (six were excluded due to missing preoperative echocardiography reports). In the whole cohort, 76 (53.1%) patients were male and 67 (46.9%) were female (Table 1). Before matching, female patients were more symptomatic (NYHA ≥ 3: 73% vs. 45%, *p* < 0.001), had higher logistic EuroSCORE (5.5 vs. 3.9, *p* = 0.006), had more urgent operations (25% vs. 11%, *p* = 0.020), MV replacements (28% vs. 11%, *p* = 0.007), secondary MR (43% vs. 16%, *p* < 0.001), and had shorter cardiopulmonary (103 min vs. 133 min, *p* < 0.001) and cross-clamp times (75 min vs. 91 min, *p* < 0.001). In terms of non-indexed LV diameters, females had significantly smaller non-indexed end-systolic (33 mm vs. 38 mm, *p* < 0.001) and end-diastolic (51 mm vs. 59 mm, *p* < 0.001) LV diameters but no significant difference in end-systolic (19.9 mm/m^2^ vs. 19.0 mm/m^2^, *p* = 0.9) and end-diastolic diameters (30.0 mm/m^2^ vs. 29.5 mm/m^2^, *p* = 0.7) when indexed to body surface area (Figure 1). PSM yielded 38 evenly matched patients for each cohort. After PSM, significant differences in non-indexed LV diameters persisted. Moreover, after PSM, females still had significantly shorter cardiopulmonary bypass (110 min vs. 126 min, *p* = 0.009) and cross-clamp times (82 min vs. 87 min, *p* = 0.010). 

### 3.2. All-Cause Mortality

**Figure 2:**
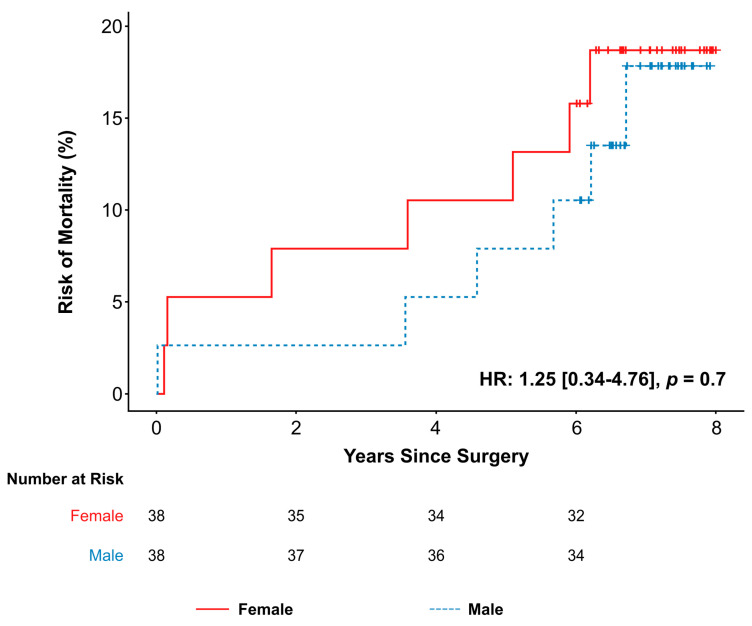
All-cause mortality by sex in PSM cohort. Hazard ratio presented as HR [95% confidence interval] and calculated using a robust sandwich variance estimate to account for the matched sets. Censoring is denoted by vertical lines. Abbreviations: HR, hazard ratio.

All patients completed follow-up with a median follow-up time of 6.67 years (IQR: 6.15–7.36). Within the PSM cohort, seven (18%) females died, and six (16%) males died. At eight years, actuarial survival was 81.3% in females and 82.2% in males (Figure 2). Mortality in females was not significantly higher than in males (hazard ratio (HR): 1.25, 95% confidence interval (CI): 0.34–4.76, *p* = 0.7). In the whole cohort, 16 (24%) females and 13 (17%) males died, which yielded no significant difference in all-cause mortality (Log-rank: *p* = 0.74) (Supplementary Figure S1). In ROC curve analysis (Table 2), LVESDi had a higher area under the curve compared to LVESD (0.565 vs. 0.545) and the cutoff of LVESDi > 19 mm/m^2^ had a higher specificity (46.0% vs. 26.7%) and comparable sensitivity (69.4% vs. 69.2%) to the cutoff of LVESD > 40 mm.

### 3.3. Length of Hospital Stay

**Figure 3:**
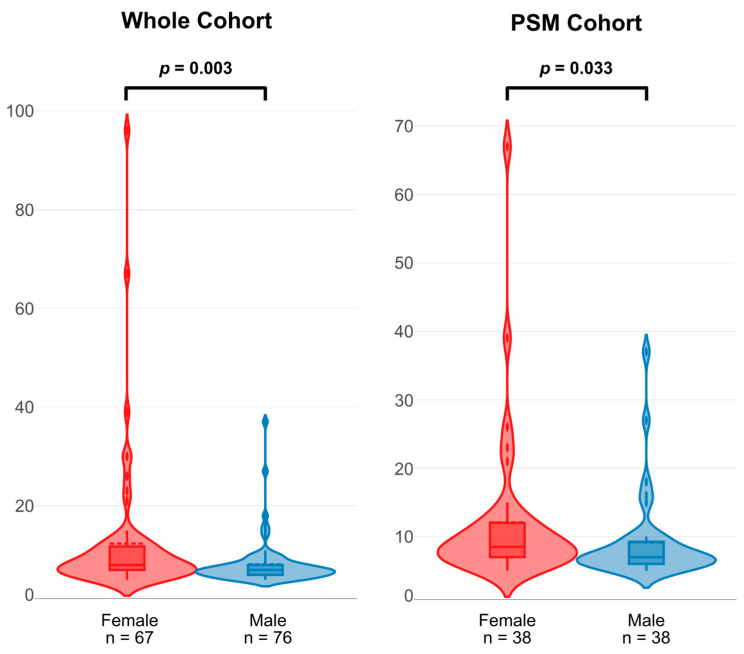
Violin plots for length of hospital stay in whole and PSM cohorts. Pairwise tests using Mann–Whitney U test. Abbreviations: PSM, propensity score-matched.

Within the whole cohort, females had a significantly longer LOS (eight days vs. seven days, *p* = 0.003). This difference remained within the PSM cohort (nine days vs. seven days, *p* = 0.033) (Figure 3).

### 3.4. Subgroup Analyses

Subgroup analyses for the primary and secondary endpoints are presented in Table 3. MV replacement was not associated with increased risk of mortality in males or females; however, MV replacement was associated with a longer LOS in males (β: 5.6, 95% CI: 4.5 to 11, *p* = 0.034) but not in females (β: 4.7, 95% CI: −4.5 to 14, *p* = 0.31). Concomitant TV intervention was associated with increased risk of mortality in both males (HR: 5.71, 95% CI: 1.20–27.2, *p* = 0.03) and females (HR: 6.80, 95% CI: 1.63–37.92, *p* < 0.01), although the risk was greater in female patients. Concomitant TV intervention did not produce a longer LOS in males or females. Finally, urgent operations were associated with increased mortality in females (HR: 4.85, 95% CI: 1.08–21.06, *p* = 0.04) but not in males (HR: 2.49, 95% CI: 0.43–11.30, *p* = 0.28) and was not associated with a longer LOS in either sex.

### 3.5. Rignt Ventricular Function

Differences in RV function between male and female patients are shown in Table 4. Within the whole cohort, female patients presented with significantly lower TAPSE (20 mm vs. 22 mm, *p* = 0.005) and had numerically lower TAPSE/PASP; however, this difference was not statistically significant (0.51 mm/mmHg vs. 0.57 mm/mmHg, *p* = 0.183). Within the PSM cohort, there were no significant differences in TAPSE, PASP, or TAPSE/PASP between males and females.

## 4. Discussion

The key findings of our study can be summarized as follows: (1) female patients are not at increased risk of mortality compared to males after MV surgery; (2) using LVESDi thresholds, as opposed to the non-indexed threshold of LVESD > 40 mm, yields improved specificity with comparable sensitivity; (3) females have longer LOS after MV surgery; (4) females and males present with significant differences in non-indexed LV diameters but this discrepancy vanishes when LV dimensions are indexed to BSA; (5) concomitant TV intervention is associated with increased mortality in both males and females, but carries a worse prognosis in females; (6) urgent operation is an independent predictor of mortality in females but not in males; (7) females may present with a greater degree of RV impairment at baseline but when matched to males do not exhibit differences in RV function.

This study adds to the growing body of literature suggesting differences in the presentation, management, and outcomes of MV surgery by sex and highlights key differences in subgroups. There have been differing results in terms of the effect sex may have on overall mortality but the main studies supporting this claim, notably an analysis of over 3700 patients [12], included patients operated on in previous decades, since which time MV operative techniques have vastly improved. Five separate studies published recently all concluded that survival after MV surgery was comparable in both sexes [1,2,6,13,14], corroborating our findings.

Most studies investigating outcomes by sex after MV intervention have had very strict inclusion criteria which may mask more subtle differences in outcomes secondary to operative techniques and presentation, which may explain the heterogeneous reports of outcomes by sex after MV intervention. In our study, the choice of replacement vs. repair did not impact mortality. Our finding that concomitant TV intervention is associated with an increased risk of mortality in female patients compared to male patients is interesting in the context of a recent study by Wagner et al. who demonstrated that women with ≥2+ TR undergoing MV surgery were less likely to receive concomitant TVr [4]. It raises the possibility that their finding may not necessarily lead to differences in outcomes between sexes, yet it remains true that concomitant TV intervention is generally underperformed for both men and women [4]. This may explain the increased risk of mortality due to TV intervention in our cohort if only those patients with extremely severe TR receive TV surgery. Evidence has also emerged that there are sex-specific phenotypes of the RV [15], raising the possibility that females may be more sensitive to the increase in afterload caused by TVr. We saw signs that, within the whole study cohort, female patients may present with a greater degree of RV impairment. However, this was not the case in the matched cohort. This suggests that baseline RV impairment may not be the driving factor behind differences in outcomes after concomitant TV intervention.

Alongside this, our data suggest that urgent operation is an independent risk factor for survival in females but not males. This factor may traditionally have been overlooked, as it is known that females tend to present with more severe disease [3,16] and thus assumed that excess mortality due to urgent operation is purely due to more severe presentations.

Our finding that females have significantly longer LOS compared to males is supported by previous studies [6]. We have shown that this is not due to inherent differences in presentation between sexes nor operative burden, especially considering that males have longer cardiopulmonary bypass and aortic cross-clamp times.

Finally, discrepancies in cardiac measurements between males and females have been widely documented, not only in LV dimensions [6,17] but also in regurgitant volume [18] and left atrial parameters [19]. Although others have shown that indexing LV dimensions to BSA provides better predictions of outcomes [17], especially in primary MR, a large study by Abadie et al. involving over 4500 patients with primary MR found that even when using indexed LV dimensions, females exhibited a lower inflection point for mortality [3]. Therefore, indexed LV measurements may not completely address the disparity in LV geometry between sexes, and absolute indexed LV end-systolic diameter cutoffs for intervention in primary MR may need to be sex-specific.

Our study has several important limitations which should be noted. The retrospective and single-center nature of this study may have introduced unknown confounders, although we sought to mitigate this using PSM. The number of patients included is small, notably in the subgroup analysis, and so our results should be interpreted with caution and require confirmation in larger, prospective studies. Moreover, core-lab adjudicated evaluation of echocardiography was not available and we did not quantify differences in anthropometrics between sexes which may influence outcomes in MR [20,21]. We also did not have data on tricuspid annular dimensions, which is an important measurement when investigating differences in TR. The long-term and retrospective nature of this study meant we were not able to discern the cause of death with sufficient precision to differentiate cardiac and non-cardiac death and so, in the interests of transparency, we elected to use all-cause mortality as the primary endpoint. Finally, we only included patients who underwent surgery and thus met guidelines for intervention; more definitive answers on differences between sexes would require a prospective evaluation of all patients presenting with MR, including those who were initially treated conservatively without surgical management.

## 5. Conclusions

In patients undergoing MV surgery for the treatment of MR, females are not at increased risk of mortality compared to males. However, concomitant TV intervention and urgent operation are associated with higher mortality in females as opposed to males. Females have significantly longer LOS compared to males, even after adjusting for differences in baseline characteristics. Finally, male and female patients have significant differences in non-indexed LV diameters which resolve after indexing to BSA alongside improving prediction specificity. Clear differences between sexes in MV surgery still exist, warranting further investigation in larger, prospective trials alongside consideration of sex-specific guidelines.

## Figures and Tables

**Table 1:** Baseline characteristics in whole and PSM cohorts by sex.

Characteristic	Whole Cohort			PSM Cohort		
	Male (*n* = 76)	Female (*n* = 67)	*p*-Value	Male (*n* = 38)	Female (*n* = 38)	*p*-Value
Clinical Characteristics						
Age (years)	68 (59, 75)	67 (54, 76)	0.9	68 (60, 75)	67 (56, 77)	>0.9
BMI (kg/m^2^)	26.4 (24.1, 29.2)	26.1 (23.6, 29.1)	0.5	25.5 (23.3, 26.9)	25.6 (22.7, 29.6)	0.9
Diabetes	6 (7.9%)	5 (7.5%)	>0.9	2 (5.3%)	3 (7.9%)	>0.9
Hypertension	36 (47%)	38 (57%)	0.3	21 (55%)	22 (58%)	0.8
COPD	6 (7.9%)	4 (6.0%)	0.8	4 (11%)	3 (7.9%)	>0.9
Atrial Fibrillation	30 (39%)	23 (36%)	0.5	12 (32%)	13 (34%)	0.8
Coronary Artery Disease	11 (14%)	6 (9.0%)	0.3	5 (13%)	4 (11%)	>0.9
Prior MI	3 (3.9%)	3 (4.5%)	>0.9	2 (5.3%)	2 (5.3%)	>0.9
Prior CVA	5 (6.6%)	9 (13%)	0.2	3 (7.9%)	4 (11%)	>0.9
Prior Cardiac Operation	2 (2.6%)	5 (7.5%)	0.3	2 (5.3%)	2 (5.3%)	>0.9
eGFR (mL/min/1.73 m^2^)	68 (57, 83)	71 (57, 80)	0.6	67 (57, 78)	72 (53, 81)	>0.9
NYHA ≥ 3	34 (45%)	49 (73%)	**<0.001**	21 (55%)	24 (63%)	0.5
Logistic EuroSCORE	3.9 (2.2, 7.1)	5.5 (3.2, 8.4)	**0.006**	4.1 (1.9, 7.2)	4.9 (3.1, 7.6)	0.2
Preprocedural Imaging						
Ejection Fraction (%)	60 (53, 65)	60 (55, 65)	0.5	60 (55, 65)	60 (55, 64)	0.5
LVEDD (mm)	59 (54, 62)	51 (45, 57)	**<0.001**	60 (56, 62)	53 (46, 58)	**<0.001**
LVEDDi (mm/m^2^)	29.0 (27.0, 32.0)	30.0 (26.0, 33.0)	0.7	29.5 (27.3, 32.8)	30.0 (26.3, 33.0)	0.9
LVESD (mm)	38 (34, 43)	33 (28, 37)	**<0.001**	38 (36, 42)	33 (28, 40)	**0.001**
LVESDi (mm/m^2^)	19.0 (17.0, 21.0)	19.0 (15.0, 21.0)	0.9	19.5 (18.0, 21.0)	18.5 (15.5, 21.0)	0.4
TR Grade ≥ 2+	23 (30%)	23 (34%)	0.6	9 (24%)	12 (32%)	0.4
Operative Characteristics						
Urgent Operation	8 (11%)	17 (25%)	**0.020**	7 (18%)	7 (18%)	>0.9
MV Replacement (vs. Repair)	8 (11%)	19 (28%)	**0.007**	8 (21%)	8 (21%)	>0.9
Concomitant TV Intervention	20 (26%)	23 (34%)	0.3	7 (18%)	11 (29%)	0.3
Secondary MR (vs. Primary)	12 (16%)	29 (43%)	**<0.001**	10 (26%)	11 (29%)	0.8
Cardiopulmonary Bypass Time (mins)	133 (111, 149)	103 (83, 125)	**<0.001**	126 (108, 146)	110 (88, 130)	**0.009**
Cross-Clamp Time (mins)	91 (81, 112)	75 (60, 90)	**<0.001**	87 (82, 111)	82 (61, 96)	**0.010**
Outcomes						
Post-Operative LOS (days) *	7 (6, 8)	8 (7, 12)	**0.003**	7 (6, 9)	9 (7, 12)	**0.033**
All-Cause Mortality	13 (17%)	16 (24%)	0.3	6 (16%)	7 (18%)	0.8

* One patient died whilst in hospital. Overall cohort characteristics are listed in Supplementary Table S1. Binary variables are presented as number (percentage) and compared with the chi-squared or Fisher’s test. Continuous variables are presented as median (interquartile range) and compared using the Mann–Whitney U test. Significant *p*-values are shown in bold. Abbreviations: PSM, propensity score-matched; BMI, body mass index; COPD, chronic obstructive pulmonary disease; MI, myocardial infarction; CVA, cerebrovascular accident; eGFR, estimated glomerular filtration rate; NYHA, New York Heart Association; EuroSCORE, European system for cardiac operative risk evaluation; LVEDD, left-ventricular end-diastolic diameter; LVEDDi, left-ventricular end-diastolic diameter indexed to body surface area; LVESD, left-ventricular end-systolic diameter; LVESDi, left-ventricular end-systolic diameter indexed to body surface area; TR, tricuspid regurgitation; MV, mitral valve; TV, tricuspid valve; MR, mitral regurgitation; LOS, length of hospital stay.

**Table 2:** Area under the curve, specificity, sensitivity, and false positive rate of LVESD and LVESDi within PPM cohort.

Variable	AUC	Specificity	Sensitivity	False Positive Rate
LVESD > 40 mm	0.545	26.7%	69.2%	73.3%
LVESDi > 19 mm/m^2^	0.565	46.0%	69.4%	54.0%

Abbreviations: LVESD, left ventricular end-systolic diameter; LVESDi, left ventricular end-systolic diameter indexed to body surface area; AUC, area under the receiver operating characteristic curve.

**Table 3:** Subgroup analysis of all-cause mortality and length of hospital stay within the PSM cohort.

All-Cause Mortality			
Subgroup	Sex	HR [95% CI]	*p*-Value
MV Replacement (vs. Repair)	Male	0.96 [0.10–4.84]	0.96
	Female	0.78 [0.08–3.71]	0.78
Concomitant TV Intervention	Male	5.71 [1.20–27.2]	**0.03**
	Female	6.80 [1.63–37.92]	**<0.01**
Urgent Operation (vs. Elective)	Male	2.49 [0.43–11.30]	0.28
	Female	4.85 [1.08–21.06]	**0.04**
**Length of Hospital Stay**			
**Subgroup**	**Sex**	**β [95% CI]**	***p*** **-Value**
MV Replacement (vs. Repair)	Male	5.6 [4.5 to 11]	**0.034**
	Female	4.7 [−4.5 to 14]	0.31
Concomitant TV Intervention	Male	5.0 [−0.62 to 11]	0.080
	Female	5.0 [−3.1 to 13]	0.22
Urgent Operation (vs. Elective)	Male	4.0 [−1.7 to 9.7]	0.17
	Female	5.2 [−4.4 to 15]	0.28

Hazard ratio presented as HR [95% confidence interval] and calculated with Firth’s penalized Cox regression. Linear regression coefficient presented as β [95% confidence interval]. Significant *p*-values are shown in Bold. Abbreviations: PSM, propensity score-matched; HR, hazard ratio; CI, confidence interval; MR, mitral regurgitation; MV, mitral valve; TV, tricuspid valve.

**Table 4:** Right ventricular function within the whole and PSM cohorts.

Characteristic	Whole Cohort			PSM Cohort		
	Male (*n* = 76)	Female (*n* = 67)	*p*-Value	Male (*n* = 38)	Female (*n* = 38)	*p*-Value
TAPSE (mm)	22 (19, 26)	20 (18, 22)	**0.005**	22 (19, 26)	20 (18, 23)	0.191
PASP (mmHg)	39 (30, 54)	34 (30, 55)	0.982	37 (30, 59)	32 (30, 45)	0.284
TAPSE/PASP (mm/mmHg)	0.57 (0.36, 0.86)	0.51 (0.37, 0.67)	0.183	0.51 (0.35, 0.86)	0.62 (0.44, 0.68)	0.888

Continuous variables are presented as median (interquartile range) and compared using the Mann–Whitney U test. Significant *p*-values are shown in bold. Abbreviations: PSM, propensity score-matched; TAPSE: tricuspid annular plane systolic excursion; PASP, pulmonary artery systolic pressure.

## Data Availability

The data supporting the conclusions of this article will be made available by the authors on request.

## Supplementary Materials

The following supporting information can be downloaded at: https://www.mdpi.com/article/10.3390/jcm14093054/s1, Supplementary Table S1: Overall baseline characteristics in whole and PSM cohorts; Supplementary Figure S1: All-cause mortality by sex in whole cohort.

## Abbreviations

The following abbreviations are used in this manuscript:

MR	Mitral regurgitation
MV	Mitral valve
TR	Tricuspid regurgitation
TV	Tricuspid valve
TVr	Tricuspid valve repair
LV	Left ventricle
BSA	Body surface area
PSM	Propensity score-matched/propensity score matching
HR	Hazard ratio
CI	Confidence interval
LOS	Post-operative length of hospital stay
NYHA	New York Heart Association
LVEF	Left ventricular ejection fraction
ROC	Receiver operating characteristic
AUC	Area under the receiver operating characteristic curve
LVESD	Left ventricular end-systolic diameter
LVESDi	Left ventricular end-systolic diameter indexed to body surface area
LVEDD	Left ventricular end-diastolic diameter
LVEDDi	Left ventricular end-diastolic diameter indexed to body surface area
