# Supplementary material for: Sex-Related Differences in Patients with Mitral Regurgitation Undergoing Mitral Valve Surgery: A Propensity Score-Matched Study
Source: J Clin Med. 2025 Apr 28;14(9):3054. doi: 10.3390/jcm14093054 (PMC12072450; doi:10.3390/jcm14093054)
Supplement: Supplementary file 1 [file jcm-14-03054-s001.zip › jcm-3595071-supplementary.pdf]

**Supplementary Table S1: Overall Baseline Characteristics in Whole and PSM Cohorts**

| Characteristic | Whole Cohort | PSM Cohort |
|---|---|---|
| | N = 143 | N = 76 |
| Clinical Characteristics | | |
| Age (years) | 67 (55, 76) | 67 (58, 75) |
| Sex (Female) | 67 (47%) | 38 (50%) |
| BMI (kg/m$^2$) | 26.1 (23.9, 29.1) | 25.5 (23.1, 28.9) |
| Diabetes | 11 (7.7%) | 5 (6.6%) |
| Hypertension | 74 (52%) | 43 (57%) |
| COPD | 10 (7.0%) | 7 (9.2%) |
| Atrial Fibrillation | 53 (37%) | 25 (33%) |
| Coronary Artery Disease | 17 (12%) | 9 (12%) |
| Prior MI | 6 (4.2%) | 4 (5.3%) |
| Prior CVA | 14 (9.8%) | 7 (9.2%) |
| Prior Cardiac Operation | 7 (4.9%) | 4 (5.3%) |
| eGFR (mL/min/1.73m$^2$) | 68 (57, 82) | 67 (56, 79) |
| NYHA ≥3 | 83 (58%) | 45 (59%) |
| Logistic EuroSCORE | 4.4 (2.3, 7.9) | 4.3 (2.2, 7.6) |
| Preprocedural imaging | | |
| Ejection Fraction (%) | 60 (54, 65) | 60 (55, 65) |
| LVEDD (mm) | 56 (50, 61) | 57 (50, 61) |
| LVEDDi (mm/m$^2$) | 29.0 (26.0, 32.5) | 30.0 (27.0, 33.0) |
| LVESD (mm) | 36 (31, 41) | 37 (32, 41) |
| LVESDi (mm/m$^2$) | 19.0 (17.0, 21.0) | 19.0 (17.8, 21.0) |
| TR Grade ≥2+ | 46 (32%) | 21 (28%) |
| Operative Characteristics | | |
| Urgent Operation | 25 (17%) | 14 (18%) |
| MV Replacement (vs repair) | 27 (19%) | 16 (21%) |
| Concomitant TV Intervention | 43 (30%) | 18 (24%) |
| Primary MR (vs Secondary) | 41 (29%) | 21 (28%) |
| Cardiopulmonary Bypass Time (mins) | 117 (98, 141) | 113 (102, 140) |
| Cross Clamp Time (mins) | 84 (70, 103) | 84 (76, 101) |
| Outcomes | | |
| Post-operative LOS (days)* | 7 (6, 10) | 8 (7, 10) |
| All-cause mortality | 29 (20%) | 13 (17%) |

Binary variables are presented as number (percentage). Continuous variables are presented as median (interquartile range).

*One patient died whilst in hospital and is not included in this figure

Abbreviations: PSM, propensity score matched, BMI, body mass index; COPD, chronic obstructive pulmonary disease; MI, myocardial infarction; CVA, cerebrovascular accident; eGFR, estimated glomerular filtration rate; EuroSCORE, European system for cardiac operative risk evaluation; LVEDD, left-ventricular end-diastolic diameter; LVEDDi, left-ventricular end-diastolic diameter indexed to body surface area; ; LVESD, left-ventricular end-systolic diameter; LVESDi, left-ventricular end-systolic diameter indexed to body surface area; TR, tricuspid regurgitation; MV, mitral valve; TV, tricuspid valve; MR, mitral regurgitation; LOS, length of hospital stay

**Supplementary Figure S1: All-Cause Mortality by Sex in Whole Cohort**

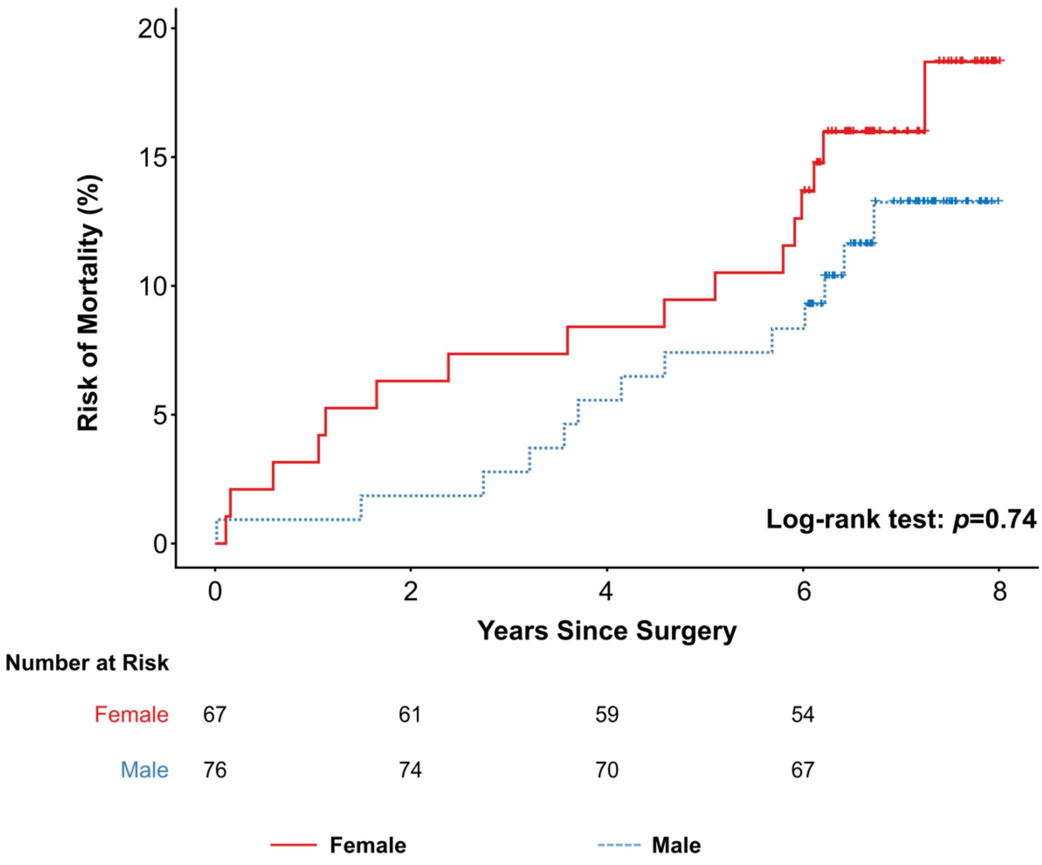


Censoring is denoted by vertical lines.
